# Comparative Prevalence of Eating Disorders in Obsessive-Compulsive Disorder and Other Anxiety Disorders

**DOI:** 10.1155/2015/186927

**Published:** 2015-08-23

**Authors:** Himanshu Tyagi, Rupal Patel, Fabienne Rughooputh, Hannah Abrahams, Andrew J. Watson, Lynne Drummond

**Affiliations:** ^1^UCL Institute of Neurology, Queen Square, London WC1N 3BG, UK; ^2^National OCD/BDD Service, Springfield University Hospital, South West London and St George's NHS Trust, London SW17 7DJ, UK; ^3^St George's, University of London, Cranmer Terrace, London SW17 0RE, UK

## Abstract

*Objective*. The purpose of this study was to compare the prevalence of comorbid eating disorders in Obsessive-Compulsive Disorder (OCD) and other common anxiety disorders. *Method*. 179 patients from the same geographical area with a diagnosis of OCD or an anxiety disorder were divided into two groups based on their primary diagnosis. The prevalence of a comorbid eating disorder was calculated in both groups. *Results*. There was no statistically significant difference in the prevalence of comorbid eating disorders between the OCD and other anxiety disorders group. *Conclusions*. These results suggest that the prevalence of comorbid eating disorders does not differ in anxiety disorders when compared with OCD. However, in both groups, it remains statistically higher than that of the general population.

## 1. Introduction

Comorbidity between eating disorders (ED) and Obsessive-Compulsive Disorder (OCD) has been recognised for over 70 years [1–3]. The strong phenomenological overlap between the two disorders has led to the descriptions of ED or its symptoms as “compulsive neurosis” [2] and “obsessive hyperactivity” [4]. Some researchers have even viewed ED as a modern expression of OCD [5]. Repeated checking [6], reassurance seeking [7], and ritualistic eating [8] seen in eating disorders can be viewed as symptoms with an obsessive-compulsive characteristic. The link between these two disorders has important implications for treatment, with outcome studies finding that those who fail to recover from ED retain high obsessionality scores, whereas, in those who recover, obsessionality scores approach those of healthy controls [9]. As well as in clinical manifestation there is also believed to be a potential biological overlap between OCD and ED, with the hypothesis that serotonin dysregulation may be common in both disorders [10–12].

Comorbidity between eating disorders and anxiety disorders more generally has been investigated, consistently finding that they frequently cooccur [13–16]. Swinbourne and Touyz [13] found that, of women presenting for treatment of an eating disorder, 65% met the threshold for at least one anxiety disorder, with 69% of these reporting the onset of the anxiety disorder preceding the onset of the eating disorder. The most common comorbid anxiety disorder to be diagnosed was social phobia (42%), followed by posttraumatic stress disorders (26%), generalised anxiety disorder (23%), OCD (5%), panic/agoraphobia (3%), and specific phobia (2%).

The same study also looked at an anxiety disorders sample, finding that 13.5% of women presenting for treatment for an anxiety disorder also met the criteria for a comorbid eating disorder, with 71% of these reporting the onset of the anxiety disorder to predate the onset of the ED. In the anxiety disorders sample, the primary disorder, for which participants with comorbid ED sought treatment, was OCD.

Despite the comorbidity of OCD and ED seeming fairly low in comparison with other anxiety disorders, empirical research demonstrates a higher statistical comorbidity of OCD and ED than expected by chance. Statistical comorbidity of OCD in patients with eating disorders has been estimated to be between 2 and forty-eight percent [17–22]. The prevalence of eating disorders in patients with OCD is estimated to be lower (8–12%) [23–25] but is again higher than expected by chance. One study [26] which systematically assessed the eating attitudes and behaviour of OCD patients using the “Eating Disorder Inventory” [27] reported that patients with OCD scored significantly higher than healthy controls on all 8 of its subscales: drive for thinness, bulimia, body dissatisfaction, ineffectiveness, perfectionism, interpersonal distrust, interoceptive awareness, and maturity fears. These results suggest that OCD patients share some of the psychopathological eating attitudes and behaviours of those with a diagnosis of ED.

The majority of research has concentrated on studying comorbid OCD in populations of patients with a primary eating disorder [28], and not the other way round. Even fewer studies have compared the prevalence of eating disorders in OCD with other anxiety disorders [25]. Exploring this association appears to be a fundamental question in order to understand potential common aetiology and to develop treatment strategies for ED within the context of OCD. In order to explore this, we conducted a prospective study with adequate power in a patient population with a validated diagnosis of a moderate-to-severe anxiety disorder. The main aim of our study was to establish and compare the prevalence rates of eating disorders in a large and well characterised sample of patients with OCD and non-OCD anxiety disorders.

## 2. Method

Our sample included all patients who were 18 years or older (*N* = 255) and assessed at a regional specialist unit for complex anxiety disorders over a period of 2.5 years (January 2008–June 2010). More details about this service are described elsewhere [29–31]. Referrals were received from general practitioners, primary care psychologists, and secondary care clinicians from southwest London (Kingston, Richmond, Merton, Sutton, and Wandsworth). All referrals were initially screened by a multidisciplinary team (MDT) of psychiatrists, therapist, and psychologists for their administrative validity against the operational criteria for this service as described as follows.

*Inclusion Criteria*

(1) All patients should be of age 18 years or more.
(2) The severity of the anxiety disorder including OCD should be in moderate-to-severe range^*∗*^ [32–34].
(3) One or more adequate trials of treatment with cognitive behavioural therapy should have proven ineffective in producing a clinically meaningful response^*∗*^ [32–34].
(4) One or more adequate trials of treatment with the first line pharmacotherapy^*∗*^ should have proven ineffective in producing a clinically meaningful response^*∗*^ [32–34],

where *∗* means as defined by guidelines issued from UK National Institute for Health and Care Excellence (NICE) [33].

All valid referrals were invited for a 90-minute face-to-face semistructured diagnostic interview. Patients unable to attend clinic based appointments due to the nature of their illness (e.g., agoraphobia: extensive compulsive behaviour centred on leaving home) were assessed at their home. Patients not fluent in English were interviewed with the assistance of an interpreter. All semistructured interviews for initial assessment were conducted within 12 weeks of the initial referral. Prior to the face-to-face meeting, relevant standardised self-report measures (described below) were sent to the patients for them to complete and bring on the day of the assessment. Semistructured interviews established the diagnosis of the primary and comorbid illnesses. These diagnoses were then validated by the MDT, before initiating the appropriate treatments.

No patients were excluded from the study due to the limitations posed by the severity of their mental or comorbid physical illness. Patients presenting with a body mass index (BMI) of less than 17 were not excluded from this assessment but were then referred for a specialist management of their weight and the underlying eating disorder.

### 2.1. Interviewing Clinicians

All clinicians who conducted the semistructured diagnostic interviews had at least 2 years' experience of assessing complex anxiety disorders, comorbid eating disorders, and OCD. Four of the clinicians were psychiatrists and the rest were fully accredited members of the British Association for Behavioural and Cognitive Psychotherapy (BABCP). All interviewers were employed by the regional specialist unit at the time of assessment. Following the diagnostic assessment, the final diagnosis was verified by the MDT of psychiatrists and accredited therapists.

### 2.2. Assessment Instruments

We used a mixture of self-report measures and clinician rated measures to minimise any self-report bias.

To screen for the presence of eating disorder, we used the SCOFF questionnaire [35], which is a standardised self-reported five-item questionnaire. This questionnaire has a high sensitivity and specificity [35] and is widely accepted as a screening tool for common eating disorders including anorexia nervosa and bulimia nervosa [35, 36]. All patients with a score of one or more on the SCOFF questionnaire were asked specific questions to rule out the presence of a comorbid or a previous ED. To ensure that the clinician had taken account of any preexisting ED, a retrospective case-note analysis was performed to confirm the presence of an eating disorder in all patients who tested positive on the SCOFF screening questionnaire. For the purpose of this study, diagnosis of a lifetime eating disorder (current or previous) was made.

The interviewing clinician rated the severity of primary and comorbid conditions using standardised clinician rated measures. The diagnosis of anxiety disorders was primarily made via the semistructured clinical interviews and verified by the MDT.

Severity of the OCD was defined by using Yale-Brown Obsessive-Compulsive Scale (YBOCS) [37]. YBOCS is standardised clinician rated instrument with 10 questions and is widely used to measure the severity of OCD [38]. It is known to have good sensitivity, specificity, and interrater reliability [37].

Comorbid depression was assessed using the self-report measure Beck Depression Inventory (BDI) [39], a clinician rated measure Montgomery and Asberg Depression Rating Scale (MADRS) [40] and formally diagnosed via the semistructured interview. BDI is a 21-item self-report questionnaire which has a good internal and external validity [41]. Montgomery and Asberg Depression Rating Scale (MADRS) is a 10-item clinician rated scale with good validity and interrater reliability [42].

### 2.3. Sample Characteristics

A total of 255 patients were identified. From this total sample, 94 patients were excluded as they did not meet the inclusion criteria as outlined previously. Our final sample included a total of 179 patients seen over a period of 2.5 years.

#### 2.3.1. Demographic Characteristics of Our Sample

All patients belonged to the same geographical area of southwest London (Kingston, Richmond, Merton, Sutton, and Wandsworth) which has a population of approximately 1 million people. 57.8% were not in any gainful employment and 67.7% were noted to be single.

#### 2.3.2. Clinical Characteristics of Our Sample

Mean age for the entire sample was 37.5 years (range 18–86, SD 13.5). 54.8% of the sample were females and 45.2% were males. 11.4% did not have any comorbid depression, 13.1% had mild depressive symptoms, 39.2% had moderate depression, and 36.4% presented with a severe comorbid depression.

**Figure 1:**
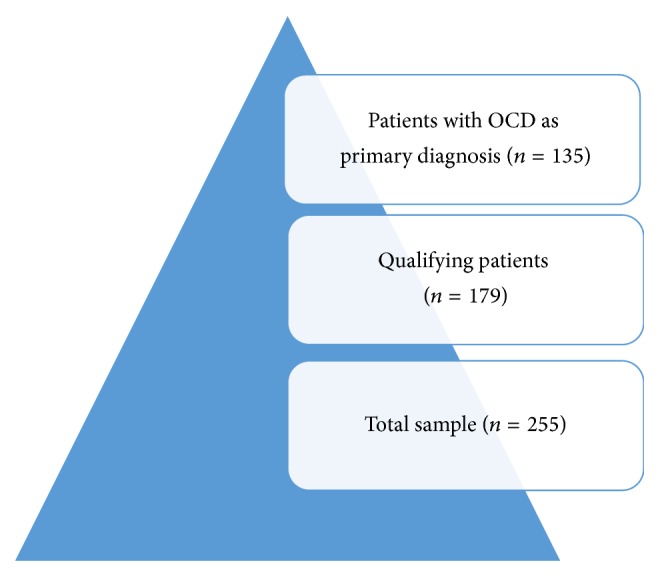


All patients in our final sample had ICD 10 defined diagnosis of an anxiety disorder. The breakdown of the primary diagnosis is described in Table 1 and Figure 1. Patients were divided into two groups based on the presence or absence of OCD. We named these groups the “OCD group” and the “non-OCD anxiety disorders group” to include all other anxiety disorders. Due to the possibility of a bias of including patients with a primary diagnosis of somatoform disorders, for example, Body Dysmorphic Disorder (BDD), all patients with comorbid BDD were excluded from the non-OCD anxiety disorders group. The most prevalent diagnosis in the non-OCD anxiety disorders group was that of generalised anxiety disorder (GAD) and social phobia. Other diagnoses in this group were agoraphobia, panic disorder, specific phobia, and mixed anxiety and depression (Table 2).

The incidence of lifetime eating disorders was calculated in both groups. As a secondary measure, we also looked at the comparative incidence of positive SCOFF scores [35, 36] (SCOFF > 1) in both groups.

### 2.4. Statistical Analysis

The data was entered into SPSS database directly from the questionnaire sheets and electronic patients' records by a clinician. All statistical analyses were completed by using SPSS version 13.0 and linear trend models were generated using Tableau desktop software. Statistical significance was calculated for the clinical and demographic differences noted between the two groups. As differences in the relative prevalence of eating disorders and anxiety disorders between the two genders can potentially skew our results, we also tested our hypothesis by doing a secondary analysis of the female population in our sample.

## 3. Results

In the non-OCD anxiety disorders group, 31.8% (*N* = 14) tested positive for a possible eating disorder on SCOFF screening questionnaire (score is 1 or more) [36]. In the OCD group, 22.9% (*N* = 31) patients had a positive result on SCOFF. However, this difference was not found to be statistically significant on Pearson's chi-square test (*p* = 0.22). This result was retested with a more robust interpretation of SCOFF (score is 2 or more) [35, 36] and it still failed to reach any statistical significance (*p* = 0.22).

ICD 10 defined clinical diagnosis of comorbid eating disorder was made in 4 patients in the OCD group and in 1 patient in the non-OCD anxiety disorders group. All 5 patients with a formal diagnosis of eating disorder were females (*p* = 0.04). As a group, females were significantly more likely to test positive on the SCOFF screening questionnaire (*p* = 0.0001), a finding which holds true even with the more robust interpretation of SCOFF, that is, SCOFF score of 2 or more.

Ritualised eating and perfectionism around food (e.g., right texture, right food items, and contamination fears) emerged as the main reasons for disordered eating in OCD for patients who did not suffer from ICD 10 defined eating disorder but still tested positive on SCOFF questionnaire.

Because it may be expected that patients with OCD in this sample have an approximately equal sex incidence [29] and patients with agoraphobia and generalised anxiety disorders have a higher incidence of higher prevalence in women, the women were compared separately. The results are summarised in Tables 3 and 4 and remained statistically insignificant (*p* = 0.72).

## 4. Discussion

Eating disorders have a considerable overlap with OCD and this may reflect common neurobiological, genetic, or psychological factors [11, 15, 19, 22, 43–50]. Some descriptive studies suggest that 50–100% of patients with ED show obsessional or compulsive features; however research in recent years have found a lower prevalence [19, 47, 49, 51, 52]. There appears to be an indication that the prevalence of OCD in ED might also be dependent on the subtype of ED [43, 50].

OCD has been considered to be a risk factor for the development of ED as its symptoms usually predate that of the ED [22]. ED subtypes might have a differential relationship with OCD as higher comorbidity with anorexia nervosa (AN) has been reported by several large studies. However, patients with bulimia nervosa (BN) with comorbid OCD are more likely to have a greater severity of ED and depression [47]. Comorbid OCD is associated with an earlier onset and prolonged duration of symptoms of ED in all subtypes of ED.

It has been suggested that the characteristics of obsessions and compulsions in ED are different from those found in OCD and are more focused on food, weight, and shape. However the research evidence to substantiate this hypothesis is lacking. The anxiolytic function of compulsions serving as an affect regulation mechanism in ED has also been considered by some researchers [53]; however such compulsive behaviours are primarily ego-syntonic and therefore distinct from compulsions seen in OCD [54].

Similarly there is an important distinction between obsessions seen in OCD and ED. Repetitive distressing thoughts about food and weight in ED are not primarily intrusive, unwanted, or meaningless in nature as in OCD [55]. However, typical OCD obsessions and compulsions can coexist in ED [56].

Therefore much of the current debate focuses on whether the obsessive and compulsive symptoms in ED are “true” OCD symptoms as seen in patients diagnosed with primary OCD. It is to be noted that, under extreme physiological stress brought about by a state of semistarvation in ED, a range of obsessional features can develop due to evolutionary mechanisms [57]. The evidence to support this view comes from studies comparing OCD symptoms in patients with varying severity of ED with more malnourished patients reporting a higher rate of concurrent obsessions and compulsions [58]. However, our study was not able to find a similar reciprocal relationship in a patient group with severe OCD.

## 5. Conclusions

Our study did not find any statistically significant relationship between the incidences of eating disorders in patients with treatment refractory OCD compared to other treatment refractory anxiety disorders. This suggests that eating disorders are unlikely to be more common in OCD when compared with non-OCD anxiety disorders group. To the best of our knowledge this is the first study to investigate comparative prevalence of ED exclusively in treatment refractory OCD patients. On comparing our results with studies which have included all patients with OCD and anxiety disorders, regardless of their status of treatment response, our finding is contrary to the existing evidence [21–26].

There may be a number of explanations for this finding. The sample for this study was limited to patients with severe OCD and anxiety disorders, refractory to treatment in primary and initial secondary care. As our sample was exclusively limited to referred patients, a selection bias in our sample cannot be ruled out. Our sample had a mean age of 37.5, an age group not associated with a higher burden of eating disorders. In addition there could be a tendency for healthcare professionals to pay more attention to the ED as this diagnosis is more likely to lead to deterioration in physical health and such patients might have been screened out before they are referred to our centre. Previous studies [20] have also shown that patients with ED and comorbid anxiety are more likely to seek help than their counterparts and this can also introduce a selection bias in our sample as it was limited to patients who are willing to seek help from a specialist centre.

## Figures and Tables

**Table 1:** Breakdown of primary diagnosis in sample.

	Number of patients	Percentage
OCD Group	135	75.4%
Non-OCD anxiety disorders group	44	24.6%

**Table 2:** Distribution of ICD 10 defined anxiety disorders in the non-OCD anxiety disorders group.

Diagnosis	Total number of patients	Percentage
Agoraphobia	5	11.6%
Social phobia	11	25.5%
Specific phobia	2	4.6%
Panic disorder	9	20.9%
GAD	11	25.5%
Mixed anxiety and depression	3	6.9%
Anxiety NOS	2	4.6%

**Table 3:** Comparative prevalence of eating disorders in both groups (entire sample, 179).

	OCD group	Non-OCD group	Statistical significance
SCOFF = 1 or more	22.9%	31.8%	ns (*p* = 0.22)
SCOFF = 2 or more	10.4%	16.3%	ns (*p* = 0.22)
ICD 10 defined diagnosis of a lifetime eating disorder	2.9%	2.2%	ns (*p* = 0.64)

**Table 4:** Comparative prevalence of eating disorders in both groups (females only).

	OCD group	Non-OCD group	Statistical significance
SCOFF = 1 or more	33.8%	42.3%	ns (*p* = 0.29)
SCOFF = 2 or more	18.3%	23.1%	ns (*p* = 0.39)
ICD 10 defined diagnosis of a lifetime eating disorder	5.6%	3.8%	ns (*p* = 0.59)
